# A virtual-reality based intervention on thoughts of the future self to reduce negative affect, depression, and suicidal ideation: protocol for a feasibility and acceptability randomized controlled pilot trial (FutureU for Mental Health)

**DOI:** 10.1186/s40814-025-01709-2

**Published:** 2025-11-15

**Authors:** Margaret A. Webb, Jean-Louis van Gelder

**Affiliations:** 1 Max-Planck Institute for the Study of Crime, Security, and Law, Rathausgasse 48, Freiburg im Breisgau, 79098 Germany; 2 Department of Psychology, George Mason University, 4400 University Dr., Fairfax, VA 22030 USA; 3 Institute of Education and Child Studies, Leiden University, Leiden, The Netherlands

**Keywords:** Virtual reality (VR), Intervention, Depression, Suicidal ideation, Episodic future thinking, Future self

## Abstract

**Background:**

Thinking about the future in a clear and detailed way is critical to daily life, generating meaning, motivation, and well-being overall. In depression and suicidal ideation (SI), executive functioning deficits can make future-oriented thinking (FT) particularly effortful. Many interventions address the quality of future thinking in depression and SI but are limited by the thoughts that a person can generate on their own. Finding ways to intervene on FT that do not rely fully on the overburdened cognitive processes of a person experiencing depression or SI may improve the efficacy of these interventions. The present pilot RCT investigates the feasibility, acceptability, and proof of concept for a novel virtual-reality (VR)-based intervention on thoughts of the future self (FutureU for Mental Health; FU-MH) to improve a person’s ability to think about the future and to reduce symptoms of depression and SI.

**Methods:**

Using a 4-month, randomized, controlled, participant-blind, add-on superiority trial with three parallel groups, we will evaluate the potential of a two-part VR-based intervention on thoughts of the future and the future self to reduce negative affect and depressive symptoms. We aim to recruit 60 participants and randomize them to either a control group, treatment group A, or treatment group B, where treatment groups are exactly the same with the exception of the order of presentation for intervention parts. Feasibility will be measured in terms of enrollment and retention, as well as subjective measures of participant engagement and embodiment in VR. Acceptability will be measured through participant self-report. Proof of concept will be evaluated through the demonstration of the intended effect on targeted cognitive mechanisms, evidence of improvement in clinical outcomes, and evidence of efficacy among those with cognitive processing deficits.

**Discussion:**

This study will provide valuable direction for further iterations of FutureU for Mental Health and a larger scale clinical trial. Implications of hypothesized outcomes include support for a novel intervention on thoughts of the future self in depression and SI, and VR as a novel medium of intervention and prevention in depression and SI.

**Trial registration:**

This trial was preregistered with OSF Registries prior to the beginning of any data collection on October 9, 2022 (doi: https://osf.io/8c5n6).

**Supplementary Information:**

The online version contains supplementary material available at 10.1186/s40814-025-01709-2.

## Background

“It is a peculiarity of man that he can only live by looking to the future–*sub specie aeternitatis*. And this is his salvation in the most difficult moments of his existence, although he sometimes has to force his mind to the task. […] The prisoner who had lost faith in the future—his future—was doomed.”—Viktor Frankl, *Man’s Search for Meaning.*

Science, philosophy, and lived experience all tell us that having faith in one’s future is critical to well-being, to meaning making, and to life itself. As Viktor Frankl describes, belief in the future is not always easy—“sometimes [one] has to force [their] mind to the task” [1]. This is especially true in depression and suicidal ideation where executive functioning deficits make future-oriented thought particularly effortful [2]. Many interventions address future thinking in depression and suicidal ideation but are limited by the thoughts that a person is able to generate on their own. Finding ways to intervene on future thinking that do not rely fully on the overburdened cognitive processes of a person experiencing depression or SI may improve the efficacy of these interventions. This is the focus of the present study, which aims to establish a proof of concept for a novel virtual reality-based intervention on thoughts of the future and the future self to reduce symptoms of depression and suicidal ideation. Virtual reality represents a promising and effective method for intervening on thoughts of the future [3, 4] but this use of VR has not yet been applied to depression and suicidal ideation.

Generating future thought involves a dynamic complex of cognitive processes which intersect with and are influenced by the cognitive processes impacted in depression [5]. Generation of detailed *positive* future thought requires attention, memory, cognitive flexibility, inhibition, and initiation. *All* of these processes are impacted in depression: attention and memory suffer from biases towards negative content [6, 7] and overgeneralization [8], thinking becomes more rigid [9], and inhibition [10] and initiation are more difficult [11].

**Fig. 1:**
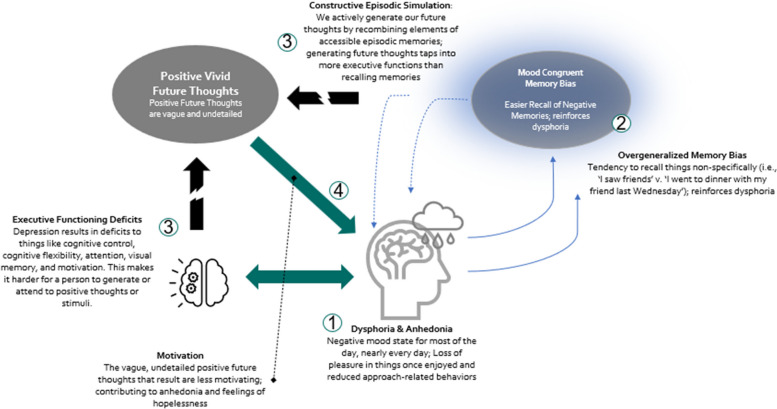
Future thinking in depression

According to the leading theory of future thought, episodic future thoughts are made up of elements of our episodic memory, flexibly recombined to create the novel imagined situation (constructive episodic simulation hypothesis [12]). For people experiencing depression, the building blocks of episodic future thoughts are overgeneralized, negative memories, and recombining these memories to generate a future thought requires a higher cognitive load than simply recalling them (see Fig. 1). Even when prompted, depressed individuals are less able to come up with positive possibilities, and any possibilities that they do generate are less vivid in their minds’ eye, which impacts the motivational power and believability of the thought [13–15]. Thus, while there are interventions, such as those cited above, that provide scaffolding for generating positive future thoughts, it is likely that the phenomenological experience of those thoughts is muted because it relies on cognitive resources that the person does not have access to.

## The potential utility of virtual reality

**Fig. 2:**
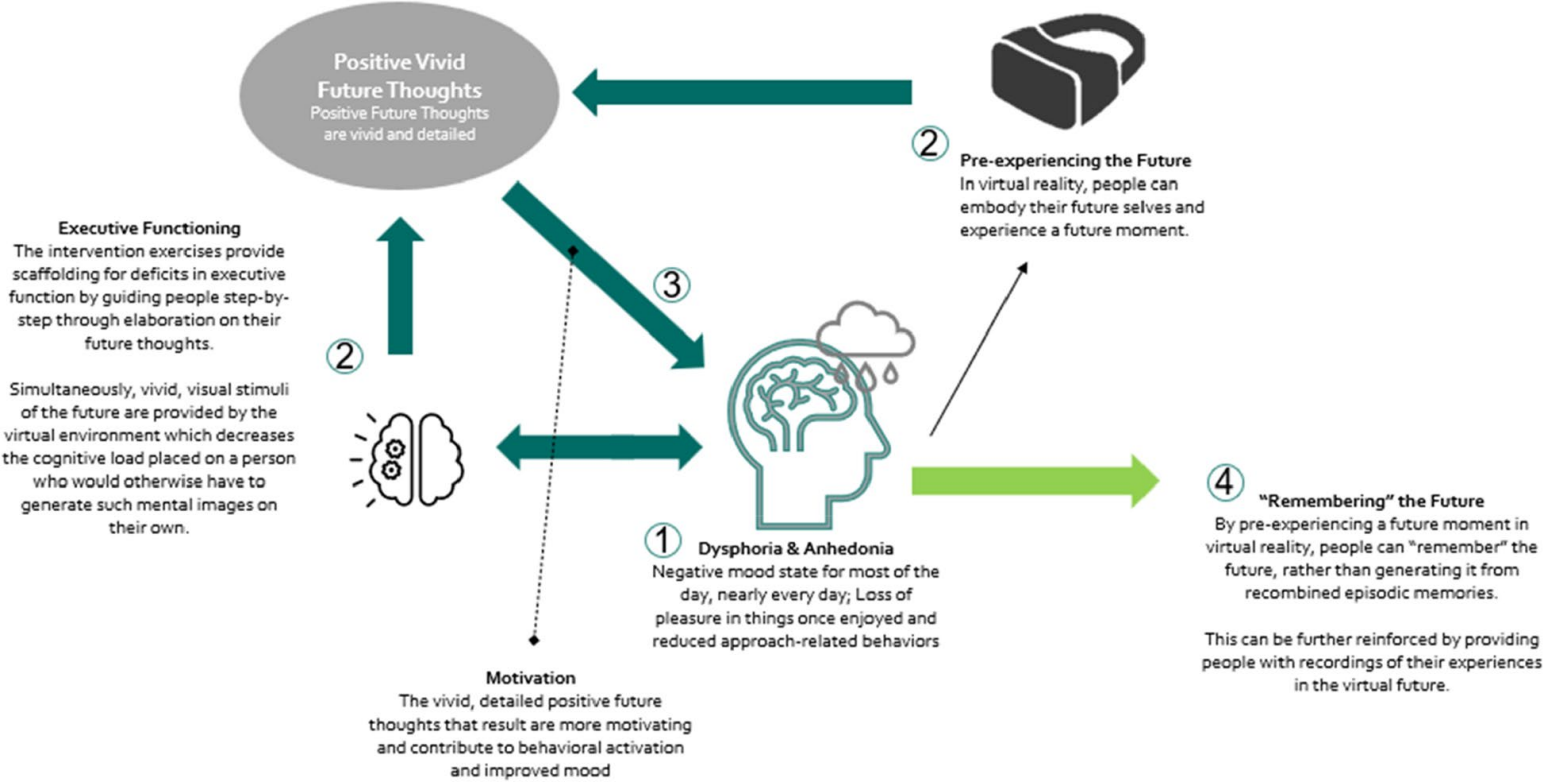
Proposed model for future thinking assisted by virtual reality

Virtual reality may provide a medium through which to activate the cognitive processes necessary to generate detailed, positive future thoughts. It may be possible that by scaffolding a person’s generation of a detailed, vivid, and positive experience of their own future through VR, they could “remember” (rather than flexibly generate) their future in a vivid and positive way, without having to rely too heavily on overburdened cognitive processes. The sensory-perceptual features of thought, such as vividness, visual details, sensory details, etc., are key to the feeling of pre-experiencing a moment [14]. These can be scaffolded and intensified by the visuals and sense of presence experienced by the person in VR in a way that traditional in-vivo methods are categorically unable to do. For instance, in VR, a person can embody and actually see themselves aged 20 years into the future, generating not only memories of the specific “future” moment during the VR exercise but also providing vivid, visual, and sensory building blocks of their future self from which to generate further future thoughts. Indeed, VR interventions focused on enhancing mental imagery have been found to deliver a stronger “dose” of the intended intervention effects than traditional in-vivo implementations, mediated by an increase in feelings of presence in VR and the vividness of the associated mental imagery [16, 17]. Figure 2 outlines our proposed model for how these advantages can play a role in more effectively intervening on depressive symptoms.

## Introduction to FutureU for mental health

There are overlapping calls to action in the clinical field which seek interventions that leverage advancing virtual reality technology [18, 19], that address the future self and episodic future thinking [20], and that consider the experience of thoughts as relevant mechanisms for intervention alongside the content of thoughts [21, 22]. The virtual reality intervention proposed in this second study, FutureU for Mental Health, answers each of these calls. FutureU for Mental Health is comprised of three parts, two of which occur in virtual reality and are the novel aspect of this mental health intervention. Each part addresses a different function of future thinking. In this study, I assess its feasibility and acceptability and establish a proof of concept.

### Coping (safety) planning and reasons to live

In the first session, participants meet with the experimenter to develop a coping plan (or safety plan, if suicidal ideation is present) and to identify things that they are looking forward to in the future that make life worth living. This kind of plan, originally developed by Stanley & Brown [23], has been deemed a gold standard approach for suicide intervention and has been demonstrated to also be effective in helping people cope with other strong, difficult emotions that are not related to suicide [24]. This session is intended to help people develop a plan to anticipate a future moment where they may be struggling emotionally and to cope with it. This strategy is a key part of many empirically based therapeutic approaches, such as cognitive behavioral therapy and dialectical behavioral therapy.

Reasons life is worth living were asked to increase participants’ future orientation, which has been demonstrated to increase coping, particularly in the face of suicidal ideation and is commonly included as an addendum to the coping plan. Participants were asked to identify as many things as they could for 1 week, 1 year, and 5–10 years into the future.

Key to the assessment of the novel VR interventions, this plan and the reasons life is worth living inform the virtual reality interventions, which are intended to build upon the coping plan and to increase and enhance future thoughts around self-efficacy in coping with difficult emotions and the motivation towards positive future outcomes.

### Self-help intervention

The goal of the self-help intervention is to increase a person’s self-efficacy around coping with difficult emotions. In this intervention, participants will first embody an avatar of themselves as they are in the present day and will sit across the table from an avatar of themselves 1 week in the future, who is at that moment experiencing difficult emotions. Here, the future self avatar has a sad expression. Participants are then asked to use their coping skills to help their future self through the difficult moment. Participants are encouraged to reference their coping plan as needed. As coping statements are given, the future avatar’s mood is seen to visibly improve.

Next, participants are asked to time travel 1 week into the future to see life through the eyes of their 1-week older future self at the moment in time that they are struggling with difficult emotions. The participant then observes their future self in the mirror, an audio recording of their coping statements is played back, and they watch as their future self’s mood improves via facial expressions. This paradigm is based on an effective self-compassion intervention, which asked individuals to give compassion to a crying child and to then receive those compassionate statements themselves [25].

First and most basically, the practice of using the coping plan is expected to increase the participant’s perceived ability to leverage it in the future and to effectively cope with difficult emotions. Next, the vivid experience of watching the future self’s mood improve with coping statements both from the third- and first-person perspective is expected to increase the participant’s ability to see themselves in the future as a person capable of feeling better despite a difficult emotion. Importantly, we expect this experimental manipulation to affect the content of these thoughts—that they think about themselves getting past difficult periods in the future—and to also affect the quality of these thoughts, e.g., the thoughts are richer in imagery and emotional valence due to the sense of pre-experiencing that virtual reality affords. Further, mental imagery related to the self occurs often in both the first- and third-person perspective, so we anticipate that both giving and receiving coping statements will contribute to enhancing these thoughts.

After leaving the session, participants will be given a recording of their coping statements and asked to listen to them at least once before the next session in 1 week’s time. In addition, they are asked to reflect on what they learned from this VR experience. The repeated exposure to their coping statements and the active reflection on their self-help experience is expected to further increase their self-efficacy in managing difficult emotions, to help participants identify the value in thinking about themselves as capable of coping, and ultimately to improve coping itself.

Importantly, we included an intervention focused on the immediate future as the function of thoughts in the immediate versus the distal future is shown to differ. Thoughts of the immediate future are more directly related to motivation and action, and function as a way to guide a person through their day-to-day activities [26]. This may be especially relevant in the context of suicidal ideation where thoughts of the immediate future are of an inability to effectively cope with difficult emotions, in direct opposition to the intended outcome of this intervention.

### Positive future memories intervention

The goal of the positive future memories intervention is to increase a person’s ability to think richly and positively about themselves and the events that will happen to them in the future. In this intervention, participants will enter virtual reality and travel through time with the therapist (experimenter) 10 years into the future. In this future therapy space, the therapist and the participant will both have been age progressed to appear 10 years older. The age progression and the virtual future environment are key pieces of the experimental manipulation of the subjective elements of thoughts of the future because they provide participants with a sensorial experience of a “future” event.

The therapist will then walk the participant through a series of exercises aimed specifically at enhancing the participant's sense of themselves in the future, both in a physical and cognitive sense. This includes a set of embodiment exercises which require the participant to observe their 10-year older self in a virtual mirror. A higher sense of embodiment in virtual reality—a higher sense that it is really *them* in the virtual environment—increases the participant’s response to emotional stimuli presented in virtual reality [27]. This portion of the intervention also allows participants to not just observe their older selves but to reflect on what they see and create a narrative around who they are in 10 years’ time.

Then, the participant will be asked to tell the therapist about a positive memory of something that happened to them in the past 10 years (this is asked from the perspective of being 10 years in the future, so all events are future events that have not really happened). The event that they are asked about will be taken from their reasons why life is worth living exercise, which was completed in session 1. Participants will be asked to use as much detail as possible to describe the event and will be asked a series of prompting questions by the therapist to help enrich the level of detail given in the description. The recall task and prompting questions were taken from the Autobiographical Interviewing task, which was specifically designed to assess and increase the richness of autobiographical memories [28]. This task has been previously adapted for use in future thinking [29]. This task is intended to provide scaffolding for generating a detailed, rich positive future thought that is of importance to the participant. The guidance provided by the therapist, coupled with the vivid visual imagery of the person’s 10-year older future self, is expected to provide participants with a positive thought about their own future that is enhanced versus what they might be able to think about on their own. This is expected to occur by providing participants with concrete building blocks for thoughts of the future that do not need to be abstractly generated. For example, following the logic of the constructive episodic simulation hypothesis [12], to think about oneself in the future without the assistance of age progression technology, a person would have to flexibly generate that image by recombining images of themselves with images they hold of other older people or knowledge they have of how aging changes features. This requires a high cognitive load. This intervention may therefore allow for the activation of such vivid positive future thoughts in a way that circumvents some of the cognitive load prerequisites.

Thoughts of the distant future have been shown to function primarily for self-definition [26, 30]. Ten years in the future was chosen as the distance of travel for this intervention to provide flexibility in capturing events that were important and potentially self-defining to the individual participant. The event need not happen *in* 10 years but would have happened *by* 10 years.

Again, participants were given a recording of their detailed description of the future event and were asked to listen to it at least once prior to the next session in 1 week’s time. In addition, participants were provided with a photograph of their age-progressed future self. Participants were also again asked to reflect on what they could take away from this VR session. As with the self-help intervention, the repeated exposure to their detailed future thought, the image of their future self, and the active reflection on their positive future memory is expected to increase their ability to think in a vivid positive way about the future—in the case of suicidal ideation, to think of a positive future beyond suicide, to increase motivation towards future goals, and to ultimately improve coping.

## Study objectives

### Primary objective

The primary objective of this study is to determine whether the intervention is feasible to administer and acceptable to participants. This objective includes an assessment of whether the FutureU for Mental Health intervention was sufficient to establish a sense of realism, i.e., engagement with the virtual environment and embodiment of the virtual avatar.

### Secondary objectives

The key secondary objective of this study is to establish a proof of concept for the FutureU for Mental Health intervention. This will be established by (a) demonstration that the intervention has the intended effect on the targeted cognitive mechanisms (i.e. enhancement of the quality of mental imagery), and (b) evidence of improvement in clinical outcomes or reduction of negative affect versus the control group, both immediately following the intervention, and 1 week and 3 months post intervention.

Another secondary objective of this study is to assess whether the virtual reality intervention is strong for those participants who experience deficits in executive functioning.

### Research hypotheses

We expect the FutureU for Mental Health intervention to be broadly accepted and feasible to implement. We expect participants to feel embodied and engaged with the virtual environment and intervention exercises.

We hypothesize that the FutureU for Mental Health (FutureU-MH) virtual reality intervention (Coping Plan plus Self-Help and Positive Future Memories interventions, collectively) will reduce negative affect, depressive symptoms, and severity/frequency of suicidal ideation, and improve mood by enhancing the quality of mental imagery in thoughts of the future and of the future self. We hypothesize this effect will continue to grow over time as participants practice a new way of thinking and cognitive patterns gradually change. Specifically, participants who receive the Coping Plan intervention plus FutureU-MH (TX: the treatment group) are expected to see an improvement in the quality of mental imagery in thoughts of the future (vividness) and of the future self (vividness, valence, connectedness, and similarity), while no change in quality of thoughts is expected for participants who receive only treatment as usual, the coping plan intervention (TAU: the control group). In turn, we expect that participants receiving TX will subsequently show a greater reduction in negative affect, depressive symptoms, and severity/frequency of suicidal ideation and a greater improvement in mood than participants receiving TAU. Similarly, participants receiving TX are expected to see an improvement in the quality of mental imagery in thoughts of the future and of the future self, while no change in quality of thoughts is expected for participants receiving TAU. These effects will be strongest at the 1-week and 3-month follow-ups.

Finally, in a more highly powered study, we hypothesize that this intervention would be most effective for individuals experiencing executive dysfunction as the images and structure provided by the virtual environment (VE) and intervention exercise will reduce the cognitive demands required to generate vivid future-oriented thought. Although we are not powered to assess this hypothesis in this pilot trial, we include the assessment measures to understand whether administration of these lengthier measures is feasible.

## Methods/design

### Study design and timeline

The intervention is designed as a randomized, controlled, participant-blind, add-on superiority trial with three parallel groups. Block randomization will occur with a 1:1 allocation. All participants will receive a Coping Plan intervention [24]. Participants only randomized to receive the Coping Plan will constitute the TAU control group. The TAU control design was chosen to ensure ethical responsiveness to the needs of all participants. Participants in each of the two treatment arms will additionally receive the FutureU-MH intervention. As the intervention is administered via two sessions each targeting different elements of the future self, treatment groups will follow an AB/BA design to allow for differentiation of session effects.

Participants will be assessed at 5 time points: pre-intervention (prior to randomization and completion of the Coping Plan), post-Coping Plan, pre- and post- each intervention session, and at both 1-week and 3 months follow-up (see SPIRIT figure in Table 1). These methods were in accordance with the Declaration of Helsinki and approved by the Ethics Council of the Max-Planck Gesellschaft.

**Table 1:** SPIRIT Figure for FutureU for Mental Health Trial

	Timepoint	− 1	0	t1	t2	**t3**	**f1**	**f2**	
Activity/assessment	Approx. time to complete	Screening and online consent	Baseline assessment	TAU: coping plan	Intervention P1 and pre-post assessments/or interim assessment 1 (Control)	Intervention P2 and pre-post assessments/or interim assessment 2 (Control)	1 week follow-up	3 month follow-up	End of study
Enrollment:									
Pre-screening consent	1 min	X							
Screening	3 min	X							
Informed consent (online)	10 min	X							
Random allocation	N/A		X						
Interventions:									
Coping plan intervention (TAU)	25 min			X					
Self-help intervention (in VR)	30 min				E *or*	E *or*			
Positive future memories intervention (in VR)	30 min				E	E			
Assessments:									
Demographics	5 min		X						
Beck Depression Inventory—II	10 min		X					X	
C-SSRS	5 min		X		X	X	X	X	
Self-efficacy	3 min		X					X	
Self-reported future self and mood	10 min		X		X	X	X	X	
Prospective imagery task	5 min		X					X	
BRIEF-A	15 min		X						
Engagement/embodiment	3 min				E	E			
Feasibility outcomes	N/A								X
Acceptability, feasibility, appropriateness	3 min				E	E			
Intervention use	1 min					E	E	E	

*TAU *treatment as usual, *VR *virtual reality,* X *all participants complete, *E *experimental groups complete, *C *control group completes

Analysis will take place at George Mason University. On the recommendation of the George Mason University Institutional Review Board, approval for analysis of the dataset will be obtained prior.

A reference guide for information outlined in the SPIRIT 2013 Checklist can be found in the supplemental materials.

### Study population

#### Inclusion criteria

1. Between the ages of 18–24, inclusive
2. Fluent in German
3. Patient Health Questionnaire −9 (PHQ-9) score > 4

#### Exclusion criteria

1. History of epilepsy
2. BDI Suicide Item = 3

##### Sample size

As this trial is primarily to establish feasibility, acceptability, and proof of concept, a full power analysis is not appropriate. However, to be able to assess feasibility process outcomes, we target recruitment at sixty participants (*n* = 60), with *n *= 40 receiving the intervention. There is no consensus on sample size of pilot studies – recommendations range from 12 [31] to 50 [32]. This sample size of *n* = 60 is in line with multiple best practice recommendations for feasibility trials and it is suggested that a sample of 30 in the intervention arm is sufficient for the main aims of this pilot study: to adequately establish feasibility and to interpret qualitative measures of acceptability [33, 34].

### Recruitment, screening and randomization

#### Recruitment, screening and randomization

All participants will be recruited from the community in Freiburg im Breisgau, Germany. Recruitment will be done via fliers placed around the city of Freiburg (i.e., on flier boards, on bicycles, etc.) and through the Max-Planck Institute’s MAXLab SONA system, which currently contains ~ 250 active participants. Fliers will be replenished around the city weekly and will include a QR code that will direct potential participants to the SONA system registration page. There, they will be directed to create an account and to take a screener questionnaire, consisting of the PHQ-9 and the BDI-II suicide item. If eligible for the study, they will immediately be directed to the online study consent form, from which they will be given a password to sign up for the first in-lab session on Qualtrics.

During the first in-lab session, participants will review the consent with the experimenter and then answer the baseline assessment battery. Following the completion of the baseline assessment, participants will be randomized to either the control condition or one of the treatment conditions. Randomization will follow a 1:1 allocation.

#### Participant timeline

A comprehensive overview of the study timeline and measure administration from screening through study completion is shown in the SPIRIT figure, Table 1.

#### Experimenter qualifications

The experimenter is a master’s level clinician who is fluent in English and German. Clinical experience and native language were prioritized when selecting an experimenter to ensure that the therapeutic elements of the study were delivered in a clinically and culturally appropriate way. The experimenter was trained by the first author on all elements of the study.

### VR equipment, environment, therapist and avatar design

#### Equipment

We will use a VIVE Pro headset to experience the VR. The experimenter will help participants adjust the headset and headphones to their comfort level. Participants will also be given controllers to interact with objects in the virtual environment. Prior to entering the VR, the experimenter will brief participants on the equipment and how to use it.

#### Environment

**Fig. 3:**
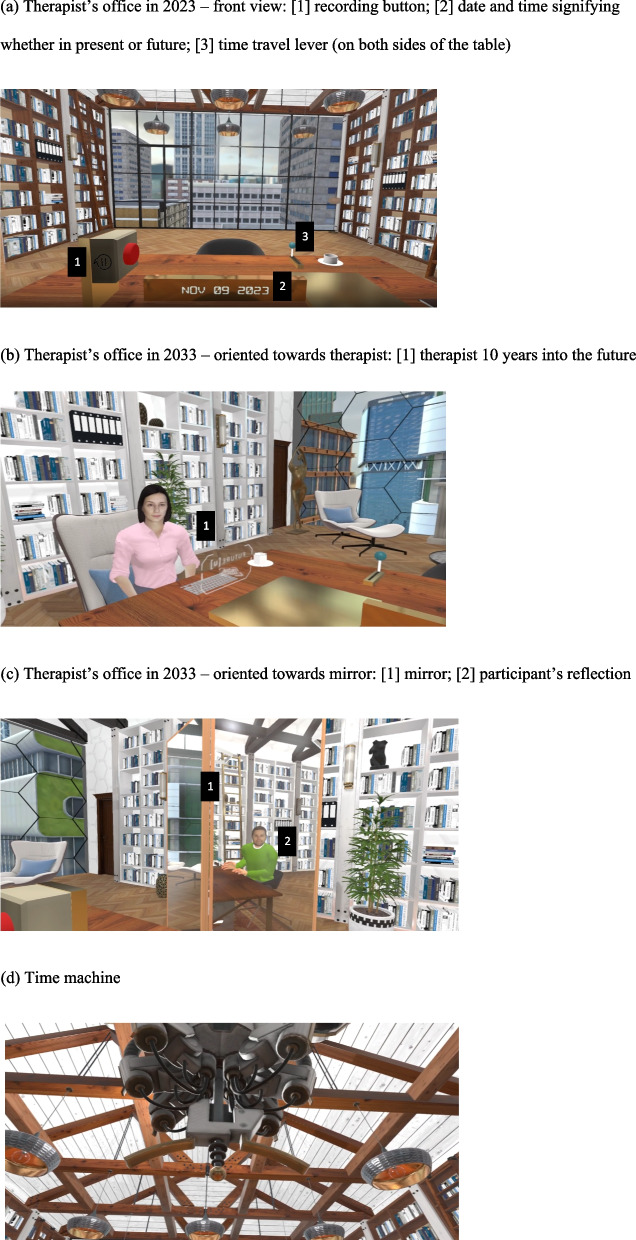
The virtual environment

The virtual environment was developed using the Unity Pro engine (version 2017.3.1f1) and resembles a therapist’s office in an urban high-rise environment. The environment changes to reflect what the office looks like in the present day and what it will look like in 10 years from now. Participants will sit at a table with the therapist sitting at one end of the table and a large mirror on the other. See Fig. 3 for visuals of the virtual environment. Above the table is a virtual time machine (the existence and function of which will be explained by the therapist while the participant is in the VR). The room in the research lab where the experiment was conducted was decorated to resemble the office in the virtual environment (i.e., a similar rug, potted plants, books, etc.) to enhance the sense of realness once in the VR.

#### Virtual therapist

**Fig. 4:**
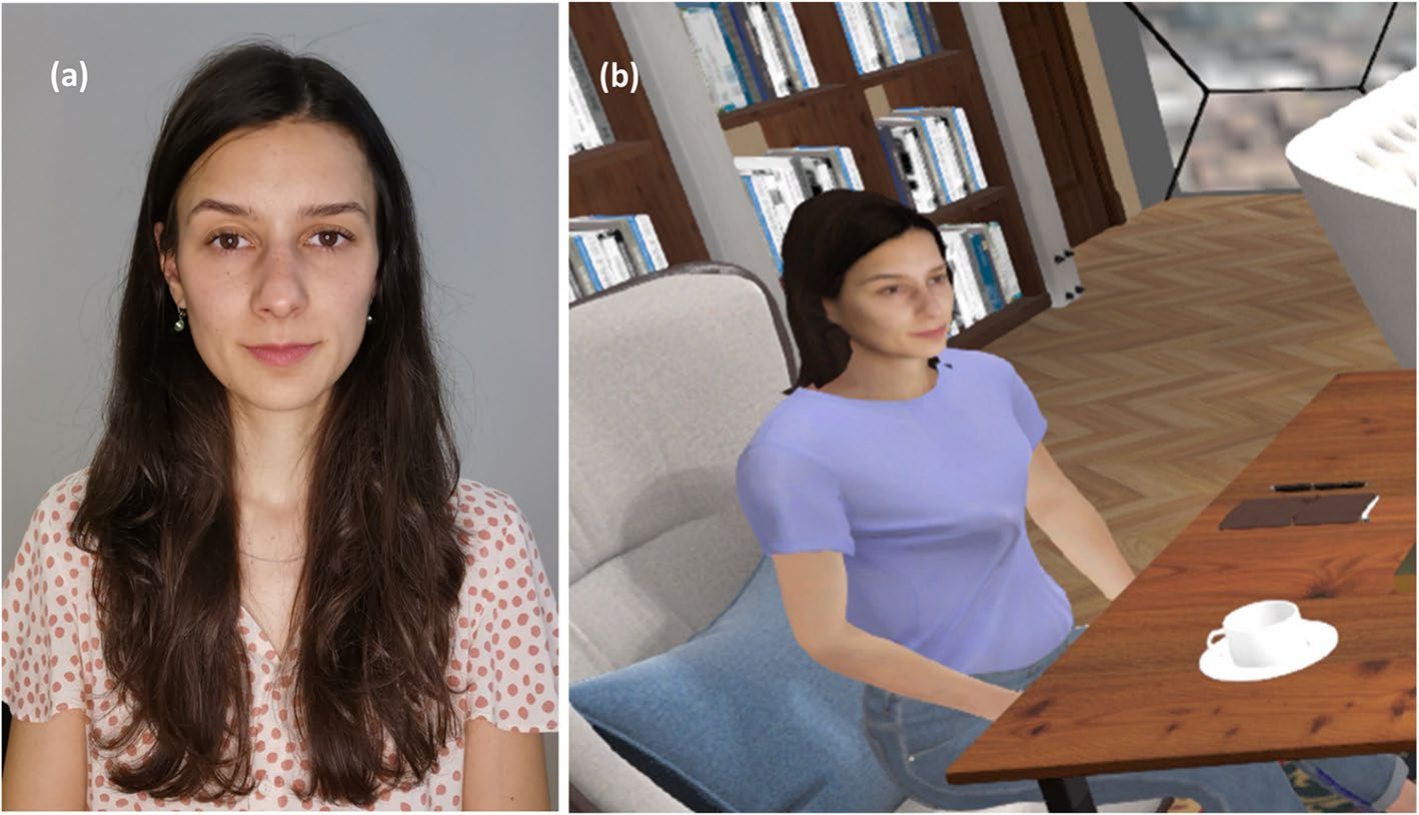
A photo of the experimenter (**a**) alongside the avatar of the virtual therapist (**b**)

The virtual therapist avatar will be controlled by the experimenter and was designed in her likeness to enhance the sense of realness for participants once in the VR – see Fig. 4. The experimenter will be the same for all participants.

##### Participant avatars

**Fig. 5:**
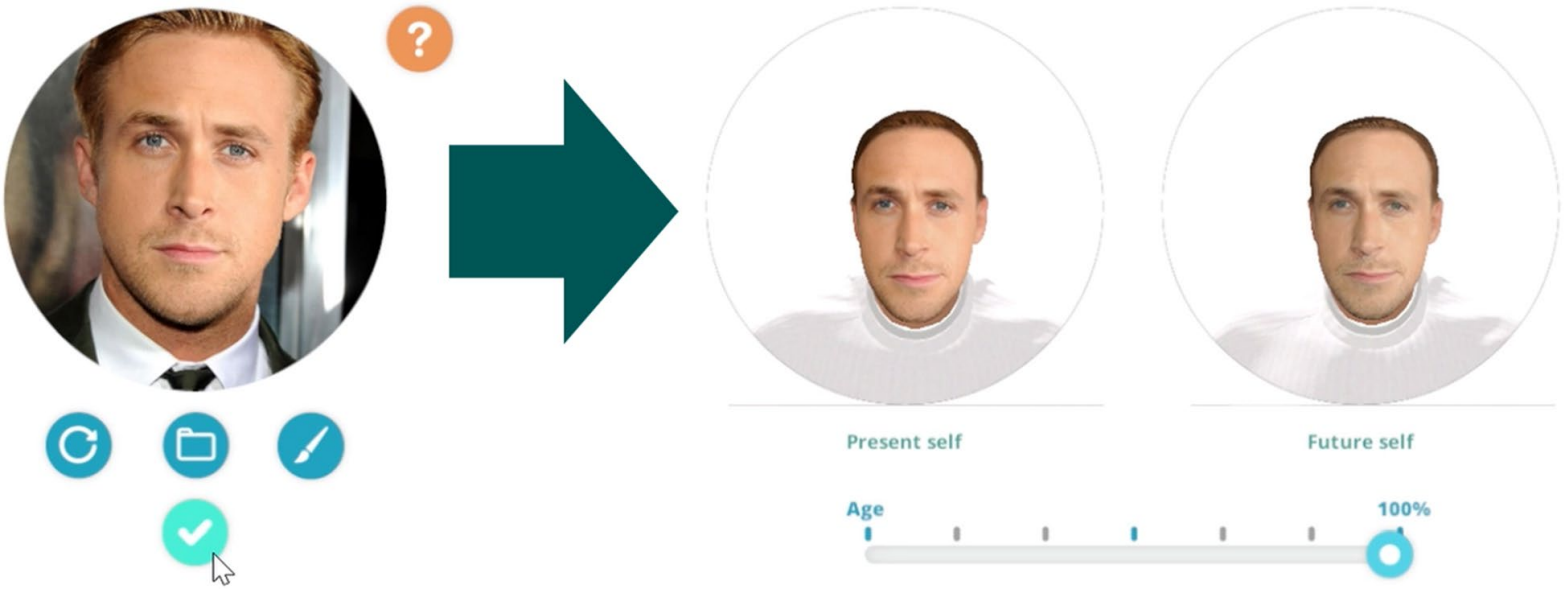
Example creation of participant avatars: Photo (left), Present Self (middle), 10-year-old self (right). The slide bar below the images allows for the adjustment of participant's aging

Personalized virtual avatars will be created for each participant. These avatars are created with a photograph taken of the participant during the first session. Avatars of the participant’s present, one-week older, and 10-year older selves are created—see Fig. 5 for an example. Aged avatars were created using plug-in software that leverages an age-progression algorithm to create a 10-year older future self (developed by Change My Face; www.changemyface.com). The avatar body is adjusted to resemble the participant’s height and physique (Note: participants are seated while in VR, so the upper body is the only aspect that they will see). The shirt color is adjusted to match the color of the clothing the participant is wearing at the time they enter the VR. Participants are asked to choose colors for their future selves to wear.

### Interventions

Three interventions will be administered. First, as a treatment as usual control (TAU), a Coping Plan is administered by the experimenter during the first in-person session to all participants. Next, participants in the treatment condition (TX) will all receive the Self-Help Intervention (SH) and the Positive Future Memories Intervention (PFM)—collectively known as the FutureU for Mental Health (FU-MH) Intervention. Participants in the TX condition will be assigned to either group A or B, which will dictate the order in which the SH and PFM interventions are received. Group A will first receive SH and then PFM; Group B will first receive PFM and then SH.

#### Coping plan intervention

The Coping Plan is an adaptation of Stanley and Brown’s [23] safety plan and is intended for use with people who struggle with regulating negative emotions but who do not report suicidal ideation [24]. The structure of the plan is the same as the Safety Plan but excludes the section on ‘Keeping the Environment Safe’. For participants who report suicidal ideation, the Safety Plan format (i.e., including ‘Keeping the Environment Safe’) will be used. The Coping Plan exercise will be informed by participants’ responses to the pre-questionnaire (specifically, the BDI-II and C-SSRS which measure depressive symptoms and suicidal ideation) and a follow-up conversation with the therapist about those responses. Participants will identify (a) Triggers and warning signs, (b) Things they can do to help themselves, (c) People or places that can distract them, (d) People they can talk to for help, (e) Professionals they can talk to for help, and (f) Things that make life worth living. A template for the coping plan can be found in Appendix 1.

#### FutureU for mental health intervention: self-help intervention

The goal of the self-help intervention is to practice coping skills and to improve self-efficacy around being able to help oneself in a moment of distress. The paradigm leveraged in this intervention is an adaptation of a self-compassion intervention developed by Falconer et al. in which participants encounter a crying child and deliver pre-rehearsed compassionate responses to the child as their current self [25]. They then embody the child and receive the compassion that they gave the child from the child’s perspective. In a pilot trial using this intervention with depressed patients, researchers found significant reductions in depression and self-criticism over a one-month period from baseline to follow-up [35].

**Fig. 6:**
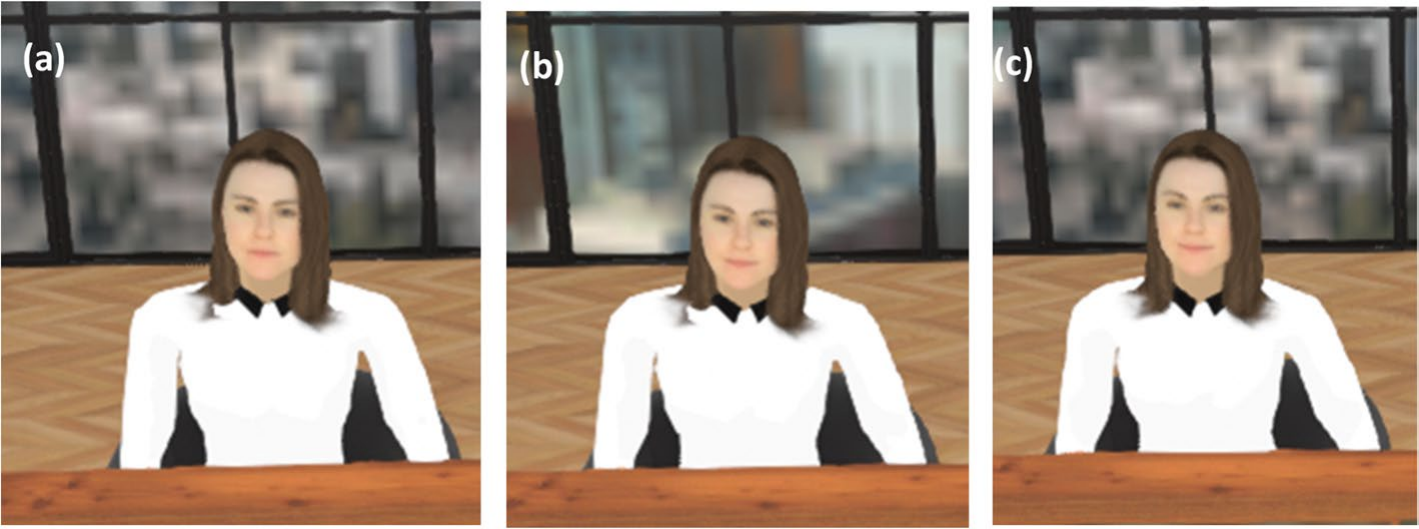
Progression of 1-week-older future self from **a** distressed to **b** neutral to **c** happy as coping statements are given

In the self-help intervention, participants will meet with a virtual ‘therapist’ who will guide them through the exercise. In the first phase of the session, the participant will be told that they are about to meet their future self (FS) at a point 1 week into the future when they are experiencing a difficult moment. Participants are instructed to think about what that moment might be like—what their FS might be thinking or feeling (here, the therapist will reference the Triggers and Warning Signs that the participant came up with during the first session). The therapist tells the participant that they will leave the room and their FS will appear in front of them. The participant is instructed that they will need to speak to their FS to help them cope with the difficult moment. To begin speaking to their FS, participants will need to press a red button that is on the desk in front of them. They are instructed that this button will also record what they are saying. Participants will be told that they may (re)record their coping statements as many times as they like until they are satisfied. A digital copy of the coping plan will be available for the participant to reference during the exercise. As the participant delivers coping statements, the FS avatar—controlled by the experimenter—will become visually less distressed (facial expressions change from distressed to neutral after 2 statements and to slightly happy after 3 + statements) – see Fig. 6.

In the second phase of the session, the participant will be told that they are now going to time travel 1 week into the future, to the exact moment in which they had just encountered their FS. This time, they will embody their FS and experience the moment through the eyes of their FS. Participants will be guided by the therapist to again try to imagine what they might be thinking and how they might be feeling at a moment when they have negative emotions. Once in the future, the participants will then listen to the recording of their own coping statements while looking at themselves in the mirror. Again, facial expressions will change from distressed to neutral to happy along with the delivery of coping statements. The participant will be given a recording of the coping statements and will be asked to listen to it at least one more time before the follow-up session. In addition, participants will be asked to reflect on what they learned from the session and how they might be able to apply it to their own lives.

#### FutureU for mental health intervention positive future memories intervention

The goal of the positive future memories intervention is to improve mood and future self-image by enhancing the valence and vividness of autobiographical future thought.

In this part of the intervention, participants will meet for a session with a ‘therapist’ in the VR environment. The therapist will be controlled by the experimenter and will guide the participant through the exercise. The participant and the therapist will both ‘time travel’ into the future and embody their future selves 10 years from the present day in a futuristic therapy office. The future therapist will then guide the participant through a series of embodiment exercises intended to enrich the participant’s perception of themselves in the future and to increase the general salience of the future-time perspective (see also [22]).

The participant and therapist will then return to the present day and debrief on how the session went and what it was like to hear about part of their future. The therapist will ask the participant to reflect on what they learned about themselves and how this exercise changed their perspective of themselves in the future. The participant will be given the image of themselves in the future as well as a recording of the conversation, which they will be asked to listen to at least one more time before the follow-up session. Participants are additionally asked to continue to reflect on their own about what they can take away from this experience.

##### Treatment fidelity

A random sample of 10% of the sessions will be recorded and assessed for adherence to the intervention manuals by the primary investigator, MW. Audio recordings will be transcribed, translated, and assessed for both adherence and competency using an adapted version of the Cognitive Therapy Scale [36].

### Outcomes measures

All measures taken during in-lab sessions will be conducted on a laptop in the lab space. All measures taken during follow-up and online control sessions will be self-administered by participants via link to a Qualtrics survey.

1. *Primary objective measures—feasibility and acceptability of the intervention*
  Feasibility: Number of eligible participants, willingness of participants to fully participate
  (i) Rates of recruitment into the study
    Number of potentially eligible participants on SONA systems.
    Number of participants who completed the PHQ-9 screener and BDI-II item.
    Number (and proportion) of participants who were eligible to participate.
    Number (and proportion) of participants who were ineligible to participate at screening and why.
    Number (and proportion) of participants who consented to participate in intervention.
    Number (and proportion) of participants who scheduled the first in-lab session.
    Number (and proportion) of participants who completed the first in-lab session.
  (ii) Retention to treatment
    Number (and proportion) of participants who successfully completed all parts of the intervention (sessions 1, 2, and 3).
    Number (and proportion) of participants who drop out of the study and, when freely shared, reasons for drop out. In accordance with our informed consent, participants are free to drop out of the study at any point without explanation.
  (iii) Completion of between session tasks
  (iv) Number (and proportion) of participants who report needing to use their Coping Plan.
  (v) Number (and proportion) of participants who report using their Coping Plan when needed.
  (vi) Number (and proportion) of participants who report listening to their Self-Help recording in the week following the intervention session and 3 months following the intervention session.
  (vii) Number (and proportion) of participants who report listening to their Positive Future Memories recording in the week following the intervention session and 3 months following the intervention session.
  (viii) Attrition at post-treatment follow-up points
    Number (and proportion) of participants who complete the 1-week follow-up questionnaire.
    Number (and proportion) of participants who complete the 1-month follow-up questionnaire.
  (ix) Levels of missing clinical outcome data
    Proportion of data completion for each questionnaire at each time-point.

Feasibility: Adherence/Compliance rates—Rating and adherence to intervention protocol. See above for details.

Feasibility: Virtual Reality Engagement and Embodiment

(i) Embodiment in Avatar
  Participants will complete a measure adapted from Banakou et al. 37 which assesses the degree to which participants experience the virtual avatar as their own body. The scale contains four items, each rated on a response scale of 1 = Strongly Disagree to 4 = Strongly Agree. Scores are determined by averaging responses to each item. Item three is reverse coded.
(ii) Proteus Effect
  Participants will complete a measure developed by van Gelder et al. 4 which assesses the degree to which people take over the characteristics associated with the avatar they embodied. The scale contains three items, each rated on a response scale of 1 = Strongly Disagree to 4 = Strongly Agree. Scores are determined by averaging responses to each item.
(iii) Presence
  Participants will complete a measure adapted from Hartmann et al. 38 to assess the degree to which participants have the feeling they were actually present in the virtual environment, i.e., a shift in users’ self-location. The scale contains four items, each rated on a response scale of 1 = Strongly Disagree to 4 = Strongly Agree. Scores are determined by averaging responses to each item.
(iv) Engagement
  Participants will complete a measure adapted from O’Brien et al. 39 to assess the degree to which the participant feels engaged in the task in virtual reality. The scale contains fourteen items, each rated on a response scale of 1 = Strongly Disagree to 4 = Strongly Agree. Scores are determined by averaging responses to each item. Items 4, 5, and 6 are reverse coded.
(v) Avatar Recognition
  Participants will complete a measure developed by van Gelder et al. 40 which assesses the degree to which participants can identify and recognize themselves in the avatar of their future self. The scale contains seven items, each rated on a scale of 1 = Strongly Disagree to 7 = Strongly Agree. Scores are determined by averaging responses to each item.

Acceptability-Feasibility: Assessment of Intervention (Acceptability of Intervention Measure—AIM; Intervention of Appropriateness Measure—IAM; Feasibility of Intervention Measure—FIM)—The AIM, IAM, and FIM are self-report measures of outcomes associated with implementation success [41]. The scales can be modified to reflect the type of service being implemented. Each scale contains four items which are rated on a response scale of 1 = Completely Disagree to 5 = Completely Agree. Scores are obtained by averaging responses for each measure. No items are reverse coded. Each scale has demonstrated strong validity and reliability [42].

Acceptability: Participant Satisfaction and general feedback—participants will respond to a brief survey assessing their satisfaction with the intervention and eliciting any feedback about the exercises. This survey was developed by the investigators for the purpose of this study.

2. *Secondary objective measures – Establishing a proof of concept for the intervention*
  Demonstrating the intended effect on the targeted cognitive mechanisms.
  (i) *Improvement in Quality of Thoughts of the Future Self –* Participants will respond to two measures assessing thoughts of the future self:
    First, a scale developed by van Gelder et al. [40] contains 5 items assessing the vividness of the future self. This scale asks participants to indicate the extent to which they agree with how clearly and easily they can describe or imagine themselves in the future. These items are on a scale of 1 = Strongly Agree to 7 = Strongly Disagree.
    For valence, a single-item pictorial assessment developed by Bradley and Lang [43] asks participants to select which of five emotional expressions ranging from sad to happy best represent how they feel about themselves 10 years in the future.
    For relatedness, two single-item pictorial assessments adapted from a task developed by Ersner-Hershfield et al. [44] which ask participants to select a pair of Euler circles which represent the extent of their feelings of connection or similarity between their present and future selves. Participants are presented with seven options ranging from completely separate circles to completely overlapping circles.
    Collectively, the vividness, valence, and relatedness items have been used to assess the broader construct of Future Self Identity [45].
    Next, a separate scale measuring the same construct, the Future Self Continuity Scale developed by Sokol and Serper [46], assesses the similarity, vividness, and positive affect for the future self. This scale contains four items related to similarity, three items related to vividness, and three items related to positive affect. Each item is rated on a scale of 1 = Not at all to 6 = Perfectly. Total and subscale scores are obtained by averaging items overall and in the respective subgroups.
    - Valence:
    - Improvement in the valence of thoughts of the future self (i.e., thoughts becoming more positive) among participants in the treatment condition from pre-to-post intervention.
    - A larger improvement in the valence of thoughts of the future self among participants in the treatment condition versus participants in the control condition from baseline to each measurement point post-intervention.
    - Vividness
      Improvement in the vividness of thoughts of the future self (i.e., the mental image becoming clearer and more detailed) among participants in the treatment condition from pre-to-post intervention.
      A larger improvement in the vividness of thoughts of the future self among participants in the treatment condition versus participants in the control condition from baseline to each measurement point post-intervention.
    - Relatedness
      Improvement in the sense of connection to the future self among participants in the treatment condition from pre-to-post intervention.
      A larger improvement in the sense of connection to the future self among participants in the treatment condition versus participants in the control condition from baseline to each measurement point post-intervention.
      Improvement in the sense of similarity to the future self among participants in the treatment condition from pre-to-post intervention.
      A larger improvement in the sense of similarity to the future self among participants in the treatment condition versus participants in the control condition from baseline to each measurement point post-intervention.
  (ii) Improvement in quality of future thoughts—Participants will complete the Prospective Imagery Task (PIT) at the baseline and final assessments [47]. The PIT asks participants to generate a mental image in response to 10 negative future scenarios and 10 positive future scenarios and indicate how vivid each image is on a scale from 1 = no image at all to 5 = very vivid.
    Improvement in the vividness of future thoughts among participants in the treatment condition from baseline to 3-months post-intervention follow-up.
    A larger improvement in the vividness of future thoughts among participants in the treatment condition versus participants in the control condition from baseline to 3-months follow-up.

*Demonstrating evidence of improvement in clinical outcomes*

(i) Improvement in symptoms of depression—Participants will respond to the Beck Depression Inventory—II (BDI-II) at baseline and at the 3-month follow-up session [48]. The BDI-II is the most widely used measure of depressive symptoms and has demonstrated good reliability and validity in clinical and nonclinical German samples [49]. The scale is comprised of 21 items assessing the presence and severity of depressive symptoms. Each item includes 4 response options ranging from no experience of symptoms to severe experience of symptoms; for example, 0 – *I do not feel sad* to 3 – *I am so sad or unhappy that I can’t stand it*. Scores are obtained by summing total items and range from 0 to 63. Total scores from 0 to 13 indicate minimal depression, 14 to 19 mild depression, 20 to 28 moderate depression, and 29 to 63 severe depression.
  Improvement in reported symptoms of depression among participants in the treatment condition from baseline to 3-months post-intervention follow-up.
  A larger improvement in reported symptoms of depression among participants in the treatment condition versus participants in the control condition from baseline to 3-months follow-up.
(ii) Reduction in frequency and severity of suicidal ideation – Participants will respond to the German language validated Columbia – Suicide Severity Rating Scale adapted for use in Research [50] at baseline, each pre-intervention questionnaire, and the 1-week and 3-month follow-up. Based on responses, participant scores will be categorized as low, moderate, or high risk.
  Improvement in reported frequency and severity of suicidal ideation among participants in the treatment condition from baseline to each measurement occurring post-intervention (i.e., session 3 pre-intervention, 1-week follow up, 3-month follow up).
  A larger improvement in reported frequency and severity of suicidal ideation among participants in the treatment condition versus participants in the control condition from baseline to each measurement occurring post-intervention (i.e., session 3 pre-intervention, 1-week follow up, 3-month follow up).
(iii) Reduction of negative affect, Improvement in Positive Affect—Participants will respond to the German language validated Positive and Negative Affect Scale (PANAS) at every measurement point [51]. The PANAS is made up of two 10-item scales measuring positive and negative affect. Each item is rated on a scale from 1 = Very Slightly, or Not at all to 5 = Extremely. Scores are obtained by averaging responses to items within the positive and negative subscales.
  Improvement in affect, i.e., increase in positive affect and decrease in negative affect, among participants in the treatment group from pre-to-post intervention and from baseline to 1-week and 3-month follow-up.
  A larger improvement in affect among participants in the treatment condition versus participants in the control condition from baseline to each measurement occurring post-intervention.
(iv) Improvement in self-efficacy—Participants will respond to the German language validated General Self Efficacy Scale (GSES) at baseline and 3-month follow-up [52]. The scale has 10 items assessing participants’ overall sense of self-efficacy with dealing with difficulties. Items are rated on a scale from 1 = Completely Disagree to 4 = Completely Agree. Scores are obtained by averaging scores across all items.
  Improvement in self-efficacy among participants in the treatment condition from baseline to 3-month follow-up.
  A larger improvement in self-efficacy among participants in the treatment condition versus participants in the control condition from baseline to 3-month follow-up.

#### Continued development of intervention procedure and protocol

In both parts of the intervention, audio recordings will be taken of the intervention targets, i.e., coping statements in SH and positive future memory in PFM. These recordings will be used post-completion of the trial to evaluate and improve the intervention procedure and protocol.

### Analyses

#### Primary objective—feasibility and acceptability of the intervention

As outlined in the outcomes section, descriptive data on the feasibility and acceptability of the intervention will be provided. Descriptive statistics will be reported for continuous and pseudo-continuous variables assessing each individual part of the intervention, i.e., for the SH and PFM interventions. Frequencies will be reported for categorical data.

**Fig. 7:**
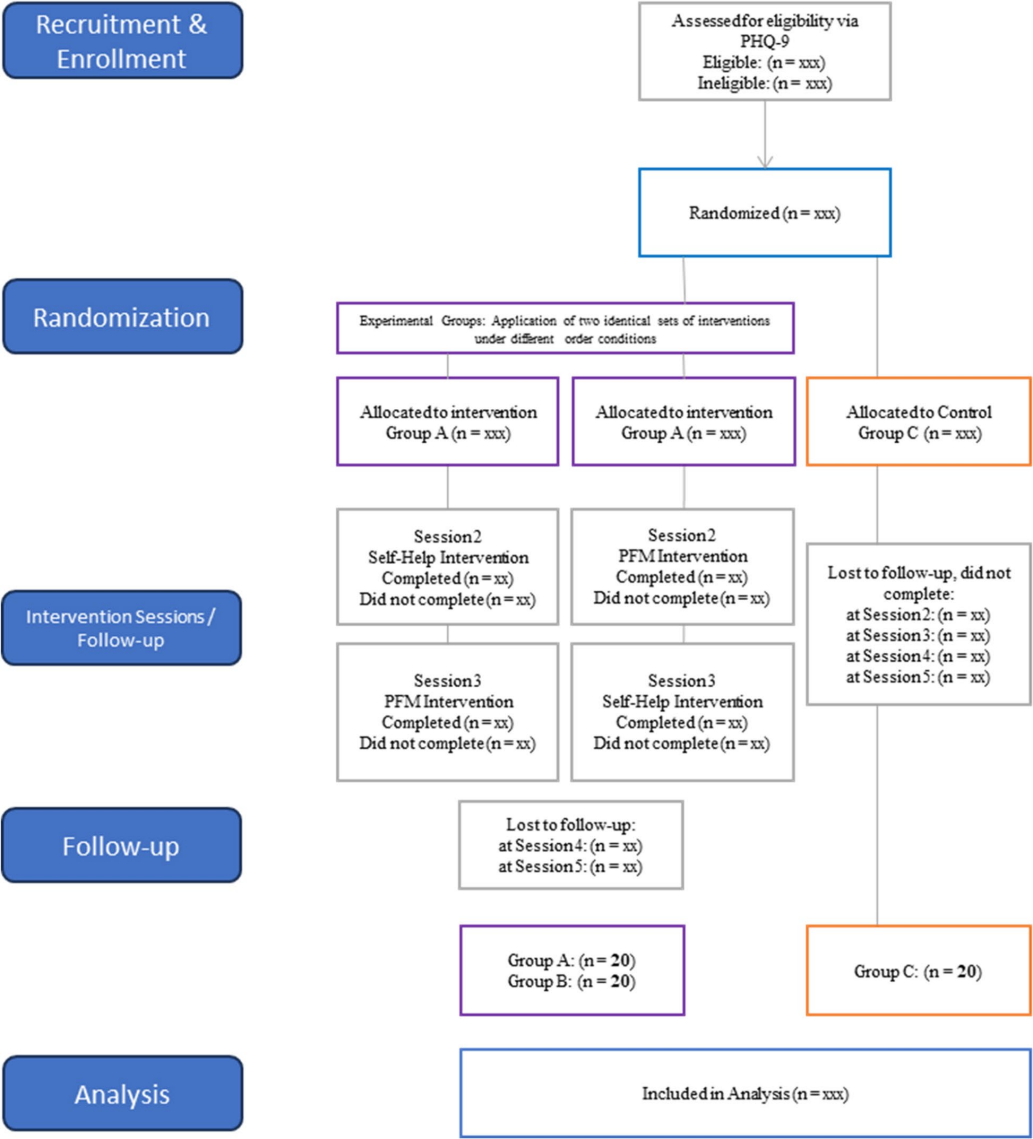
CONSORT Flow Diagram for FutureU for Mental Health Trial

Participant’s progress through the trial will be reported in a CONSORT diagram*—*see Fig. 7. This will indicate the number of participants who were eligible, consented, began, and completed the study, as well as the number of participants who dropped out, at which timepoint, and why (if reason is known).

#### Secondary objective—establishing a proof of concept for the intervention

Assessment of change in targeted cognitive mechanisms and clinical outcomes will be completed as is outlined in the outcomes section. Due to the modest sample size, these analyses will be completed to pilot the method for a full-scale RCT and to explore trends. The significance of effects will be interpreted and discussed within the context of the statistical limitations of a pilot trial.

Between-group differences for participants in the treatment condition versus the control condition will be assessed using independent-sample *t*-tests. Here, interpretation of Cohen’s *d *will be done using between-group standard deviations. The significance of between-subject changes will also be assessed through calculation of a reliable change index (RCI) [53] and evaluation of confidence intervals for mean differences.

Between-group differences in the targeted cognitive mechanisms and clinical outcomes will additionally be assessed through Latent Growth Curve Modeling (LGCM). This analysis will allow us to evaluate the difference in trajectories of change in cognitive mechanisms and clinical outcomes between the control and treatment conditions. The hypothesized changes in thought processes and subsequent clinical outcomes are expected to continue to grow over time post-intervention, rather than improve wholly and discretely at the point of intervention. The LGCM approach will allow us to observe whether this growth hypothesis is borne out.

For all analyses, age and gender will be included as covariates. Gender is included for the potential biasing effect on reports of depression. Cross-nationally, women endorse depression at highly elevated rates versus men [54]. Age is included for the potential biasing effects on future-oriented thinking. The future is conceived differently at different stages of life. Although variation in stages of life is comparatively low given the already constricted age range (18–24), there are still some meaningful differences in life stage that may impact future perspective when comparing an 18 versus 24-year-old.

### Progression criteria

Decisions regarding whether and how to proceed with testing this intervention on a larger scale are based on the feasibility and acceptability outcomes outlined above. Progression criteria are described in Table 2, following best-practice recommendations for progression criteria reporting [55].

**Table 2:** Progression criteria

	Go: proceed with RCT	Amend: proceed with changes	Stop: do not proceed unless changes are possible
Feasibility of participant recruitment Can 60 participants be recruited who meet eligibility criteria (20 per group)?	If ≥ 12 participants can be recruited per month, 60 total	If ≥ 8 participants can be recruited per month, 40 total	If no participants can be recruited per month
Feasibility of participant retention Can ≥ 85% of participants be retained in the study until completion?	≥ 85% (95% CI*: 74–92%) retained through session 4 (1 week follow-up)	≥ 60% retained through session 4 (1 week follow-up)	< 60% retained through session 4 (1 week follow-up)
Intervention implementation Based on qualitative and quantitative data from both participants and experimenter	Delivery of intervention judged strongly feasible	Delivery of intervention judged feasible	Delivery of intervention judged possibly feasible
Feasibility of between-session tasks	≥ 60% participants complete between-session tasks	≥ 40% participants complete between-session tasks	< 40% participants complete between-session tasks
Feasibility of virtual reality medium (embodiment and engagement)	≥ 85% participants report acceptable ratings for avatar embodiment, engagement, and recognition	≥ 60% participants report acceptable ratings for avatar embodiment, engagement, and recognition	< 60% participants report acceptable ratings for avatar embodiment, engagement, and recognition
Acceptability of intervention Based on qualitative and quantitative data from both participants and experimenter	Intervention judged highly acceptable	Intervention judged acceptable	Intervention judged potentially acceptable

*Wilson interval calculated based on a sample of *n *= 60

## Ethics and dissemination

### Adverse event reporting

As the trial is conducted, any serious adverse events (SAE; all serious undesired events independent of whether they have a suspected relationship to the intervention) or suspected unexpected serious adverse reactions (SUSAR; a serious adverse event which is suspected to be related to some part of the intervention) will be recorded. Any SUSAR will be reported to the ethics committee within 15 days of the investigators becoming aware of the event. After two SAE/SUSAR in a row, the trial will be paused and continuation will be evaluated.

### Safety protocol

Participants are asked to answer questions regarding suicidal ideation, as such a safety protocol was developed to ensure that any participant who presents as at risk for suicide receives adequate care. As a part of the study procedure, participants will respond to a self-report version of the C-SSRS for research. Immediately following completion of the questionnaires, the experimenter—who is a trained therapist—will review participant responses and confirm answers to the C-SSRS, using the full baseline/screening version of the measure. If a participant reports suicidal ideation, the experimenter will review the additional suicidal ideation intensity questions and suicidal and nonsuicidal self-injury behavior items with the participant. Following a review of participant answers, the experimenter will then complete a Safety Planning intervention with the participant in lieu of the Coping Plan intervention. The Safety Planning intervention is currently the best practice for responding to suicidal ideation. Participants will be given a copy of the plan to take home and (all participants) will be provided with a resource sheet of local and national mental health resources. Participants will be encouraged to consult their plan and the resource sheet. In the case of suicidal ideation, participants will be offered consultation with a licensed clinical psychologist, who is consulting on the study. In the case of significant risk (as deemed by the experimenter), the licensed clinical psychologist will be contacted for consultation on how to proceed. All risk procedures will be documented in the participant’s study file. The experimenters will not be made aware of whether participants chose to pursue subsequent mental health services. In the unlikely case of an acute mental health crisis in the lab, participants are informed through the consent that the experimenter may call emergency services to ensure they receive the best care possible.

### Data safety and participant confidentiality

All data collected will be stored and scientifically analyzed in accordance with data protection laws (EU General Data Protection Regulation, Federal Data Protection Act [German Bundesdatenschutzgesetz]).

Participant responses will be completely anonymized from the beginning of the study. Upon consenting, participants are given a participant ID, and this number will be used to identify and group their data across timepoints. Responses are never directly linked to participant names or other identifying information. All anonymized data will be stored in a password-protected file on an encrypted server. Recordings, which may be considered to be personal identifiers, will be stored separately from the anonymized data in a password-protected file on an encrypted server. Participants’ paper files—i.e., a copy of their coping plan, the C-SSRS follow-up, any other notes – will be stored securely in a locked filing cabinet in a locked office in the lab. Only the investigators and experimenter will have access to any of these data files. All data will be backed up onto two password-protected and encrypted hard drives, which will be kept in locked cabinets in separate offices belonging to the investigators.

Participants will be offered a copy of the recordings taken during the sessions to take home with them, which they are free to refuse. Participants are made aware through the informed consent and reminded during the session that if they choose to take home the recording, they assume responsibility for keeping it private.

#### Modification and dissemination

Any modifications made to the intervention will be reported to the ethics committee promptly. It is intended that the results from this trial will be reported and disseminated locally, at the institute level, as well as at national and international conferences, and through publication in scientific journals. This trial is intended to inform further iterations of the intervention and the potential and procedure of a subsequent full-scale RCT.

#### Potential implications

This study has implications in both clinical and cognitive psychology. To our knowledge, this is the first study to investigate the potential use of virtual reality as a therapeutic tool in a population of individuals with depression and the first study to investigate the utility of virtual reality as a medium through which to enhance the subjective quality of future thought in any clinical population.

The findings have the potential to inform the development of novel interventions for depressed and suicidal young adults and novel prevention techniques for young adults at risk for developing depression and suicidal ideation. This study will provide evidence as to the feasibility and acceptability of implementing a virtual reality-based intervention among young adults experiencing depression.

Further, although this pilot study is not powered to detect effects of clinical outcomes, results will guide the development of a full-scale RCT. Results of a well-powered study will provide valuable insights on the cognitive processes (e.g., executive functioning, remembering, etc.) involved in future thinking and evidence as to whether virtual reality can provide scaffolding for deficits in those cognitive processes among individuals with depression.

## Supplementary Information

Supplementary Material 1.

## Data Availability

Not applicable.

## Abbreviations

AIM: Acceptability of Intervention Measure
BDI-II: Beck Depression Inventory-II
BRIEF-A: Behavior Rating Inventory of Executive Function for Adults
CONSORT: Consolidated Standards of Reporting Trials
C-SSRS: Columbia–Suicide Severity Rating Scale
FIM: Feasibility of Intervention Measure
FS: Future Self
FU-MH: FutureU for Mental Health
GSES: General Self-Efficacy Scale
IAM: Intervention Appropriateness Measure
LGCM: Latent Growth Curve Modeling
OSF: Open Science Forum
PANAS: Positive and Negative Affect Scale
PFM: Positive-Future Memories Intervention
PHQ-9: Patient Health Questionnaire-9
PIT: Prospective Imagery Task
PS: Present self
RCI: Reliable Change Index
RCT: Randomized controlled trial
SAE: Serious adverse event
SH: Self-help intervention
SI: Suicidal ideation
SPIRIT: Standard Protocol Items: Recommendations for Interventional Trials
SUSAR: Suspected Unexpected Serious Adverse Reactions
TAU: Treatment as Usual Control Group
TX: Treatment Group
VE: Virtual environment
VR: Virtual reality
