# Supplementary material for: A virtual-reality based intervention on thoughts of the future self to reduce negative affect, depression, and suicidal ideation: protocol for a feasibility and acceptability randomized controlled pilot trial (FutureU for Mental Health)
Source: Pilot Feasibility Stud. 2025 Nov 15;11:143. doi: 10.1186/s40814-025-01709-2 (PMC12621405; doi:10.1186/s40814-025-01709-2)
Supplement: Supplementary file 1 — Supplementary Material 1. [file 40814_2025_1709_MOESM1_ESM.docx]

| Recognizing Warning Signs and Vulnerabilities | Things I Can Do to Support Myself in the Moment | People and Places that Can Distract Me | People Who Support Me/People I Can Ask for Help | Professionals I Can Ask for Help |
| --- | --- | --- | --- | --- |
| *Thoughts, feelings, or actions that you or others notice when you feel distressed* | *Options to distract yourself when you’re alone* | *Where to go and who to talk to for distraction* | *Contacting family members or friends for help (at least one adult)* | *Who I can ask for help* |
|  |  |  |  |  |

| **My reasons to live *(Things that are most important to me and worth living for):*** *1 Week: 1 Year: 10 Years:* |
| --- |

| **Warnzeichen und Auslöser erkennen** | **Dinge die ich tun kann, um mich selbst zu unterstützen** | **Menschen und Orte, die mich ablenken können** | **Personen die mich unterstützen oder die ich um Hilfe bitten kann** | **Fachleute, die ich um Hilfe bitten kann** |
| --- | --- | --- | --- | --- |
| *Gedanken, Gefühle oder Handlungen, die Sie oder andere bemerken, wenn es Ihnen nicht gut geht* | *Möglichkeiten, sich selbst zu helfen* | *Orte wohin Sie gehen können und Personen mit denen Sie sprechen können, um sich abzulenken* | *Familienmitglieder oder Freunde, die Sie um Unterstützung bitten können* | *Fachleuchte an die Sie sich wenden können* |
|  |  |  |  |  |
| **Worauf ich mich freue - *Gründe, warum das Leben lebenswert ist*:**  ***1 Woche:                                                                                          1 Jahr:                                                                                    5-10 Jahre:*** | | | | |
